# The Use of Extracorporeal Shock Wave Lithotripsy to Manage a Knotted Ureteric Stent: A Case Report

**DOI:** 10.7759/cureus.69626

**Published:** 2024-09-18

**Authors:** Hassan A Al Dehneen, Ahmed R Alfarhan, Abdulrahman A Alhoziem, Mohammed S Albarrak, Wassel A Aleisa, Ali A Alsamin

**Affiliations:** 1 Urology, Almoosa Specialist Hospital, Hofuf, SAU; 2 Urology, Prince Saud Bin Jalawi Hospital, Hofuf, SAU; 3 Urology, King Fahad Hospital, Hofuf, SAU; 4 College of Medicine, Imam Abdulrahman Bin Faisal University, Dammam, SAU

**Keywords:** ct kub, dj stent, extracorporeal shock wave lithotripsy (eswl), holmium:yag laser, lower urinary tract symptoms (luts)

## Abstract

This case report describes the successful management of a knotted ureteric stent in a 57-year-old male with diabetes mellitus, who presented with left flank pain and lower urinary tract symptoms after seven months of stent placement. Initial imaging revealed migration and encrustation of the stent, along with knotting at the proximal end. As rigid cystoscopy to attempt stent retrieval met resistance, a semirigid ureteroscope was used to laser transect the stent, allowing for the insertion of a new stent. Following this, extracorporeal shock wave lithotripsy was performed to target the knotted portion, successfully facilitating its unknotted migration. Final imaging confirmed the appropriate positioning of both stents, enabling their subsequent removal without complications. This case underscores an effective, innovative approach to managing complex ureteric stent complications.

## Introduction

Since the ureteric stent was first described in 1967 by Zimskind et al., it is widely used for different indications [1,2]. It is indicated to be used in case of pain related to ureteral obstruction due to any cause, diversion of urine, or urine drainage post-endourological procedures [3]. The decision to insert a ureteric stent may be complicated by infection in the urinary tract, stone, stent encrustation, or fragmentation [4]. Another extremely rare complication mentioned in the literature is the knotting of ureteric stent [5], with fewer than 30 cases reported according to a case report published in June 2020 [6]. Management of this issue can be approached by several techniques as mentioned in the literature, such as fine endoscopic grasping, guidewire assistance removal, and retrograde or antegrade ureteroscopic extraction [7].

Here, we report a case of an unusual way of management by exposing the patient to a session of extracorporeal shock wave lithotripsy (ESWL) before smooth extraction by ureteroscopy.

## Case presentation

**Figure 1:**
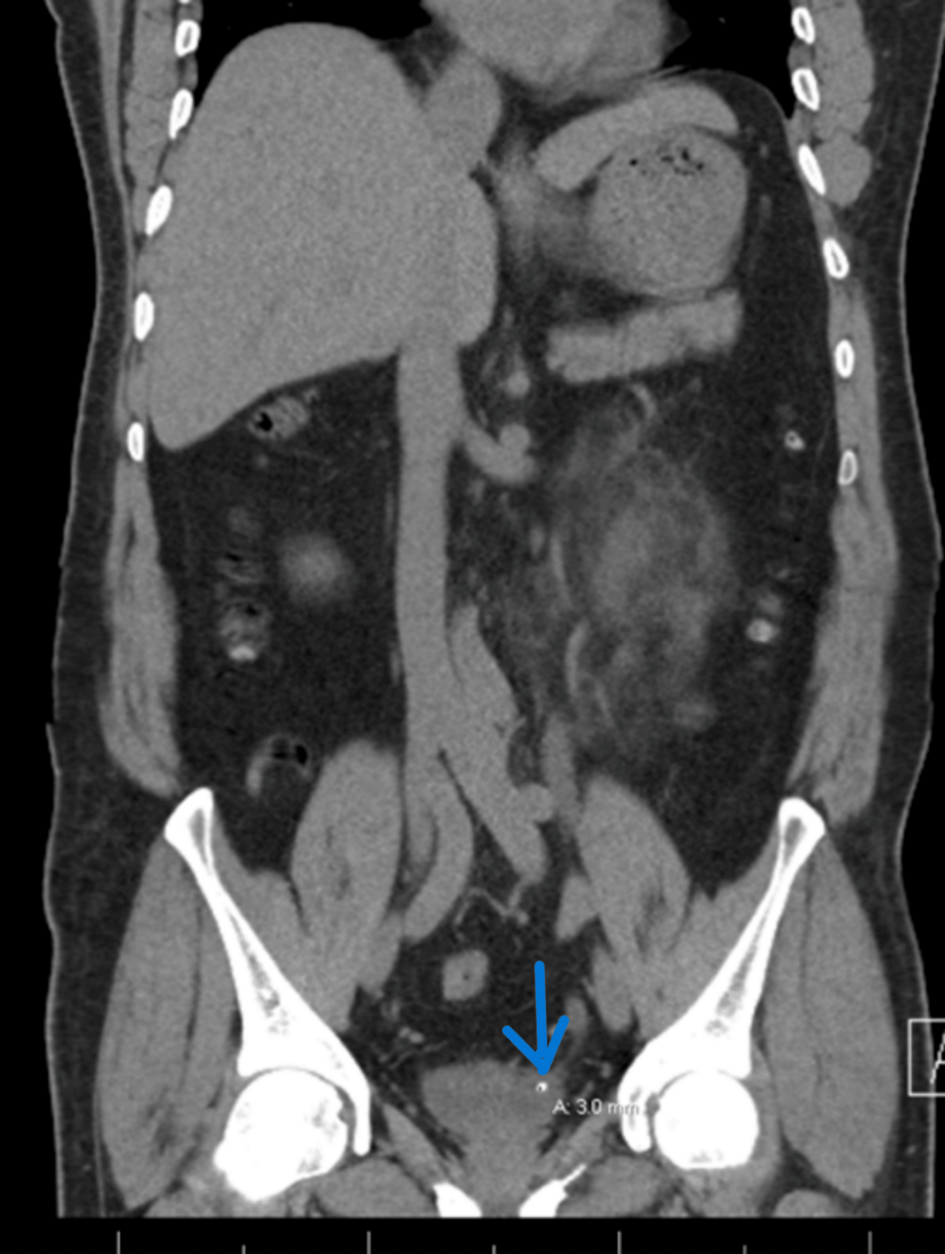
A 3 mm stone at the left vesicoureteric junction associated with extensive perinephric fat stranding of the left kidney with ureterohydronephrosis.

A 57-year-old male (height: 172 cm) with diabetes mellites presented to the emergency department complaining of left flank pain with lower urinary tract symptoms, no history of vomiting, afebrile for three days, and impaired kidney function. A computed tomography scan of the kidneys, ureters, and bladder (CT KUB) without contrast showed a 3 mm stone at the left vesicoureteric junction associated with extensive perinephric fat stranding of the left kidney with ureterohydronephrosis (Figure 1).

**Figure 2:**
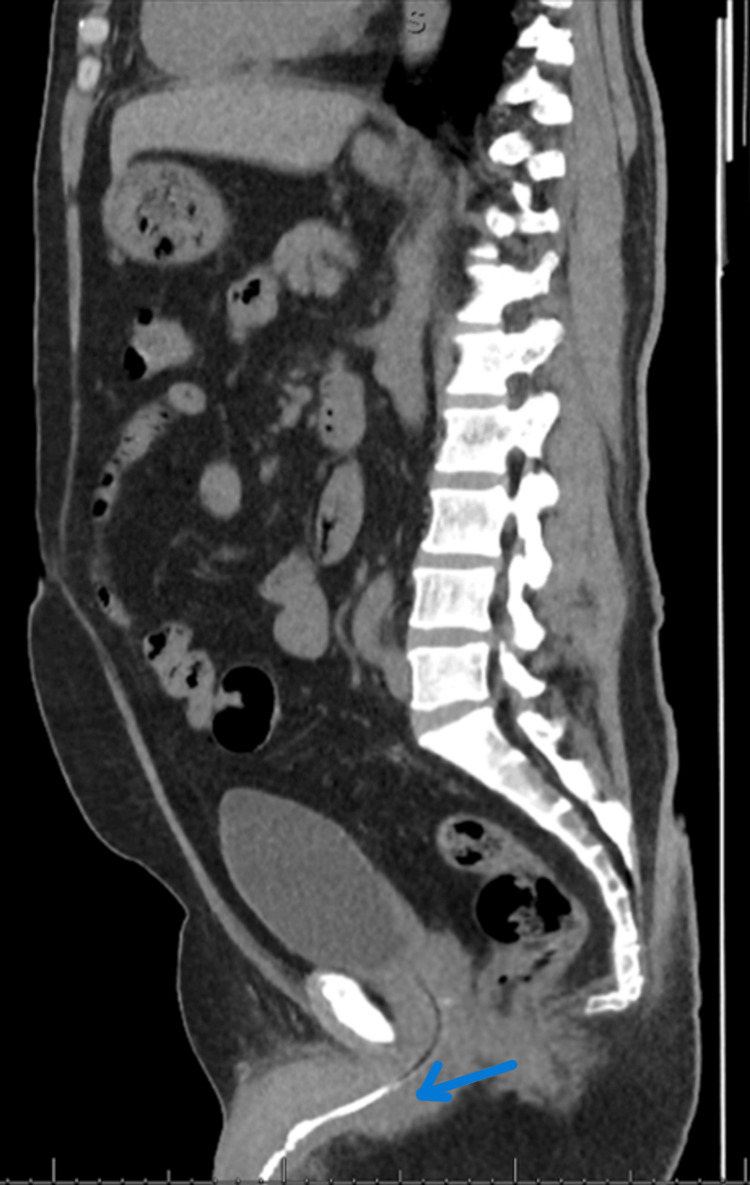
The lower part of the left double J ureteric stent can be seen migrated to the proximal part of the anterior urethra with encrustation around the lower part.

**Figure 3:**
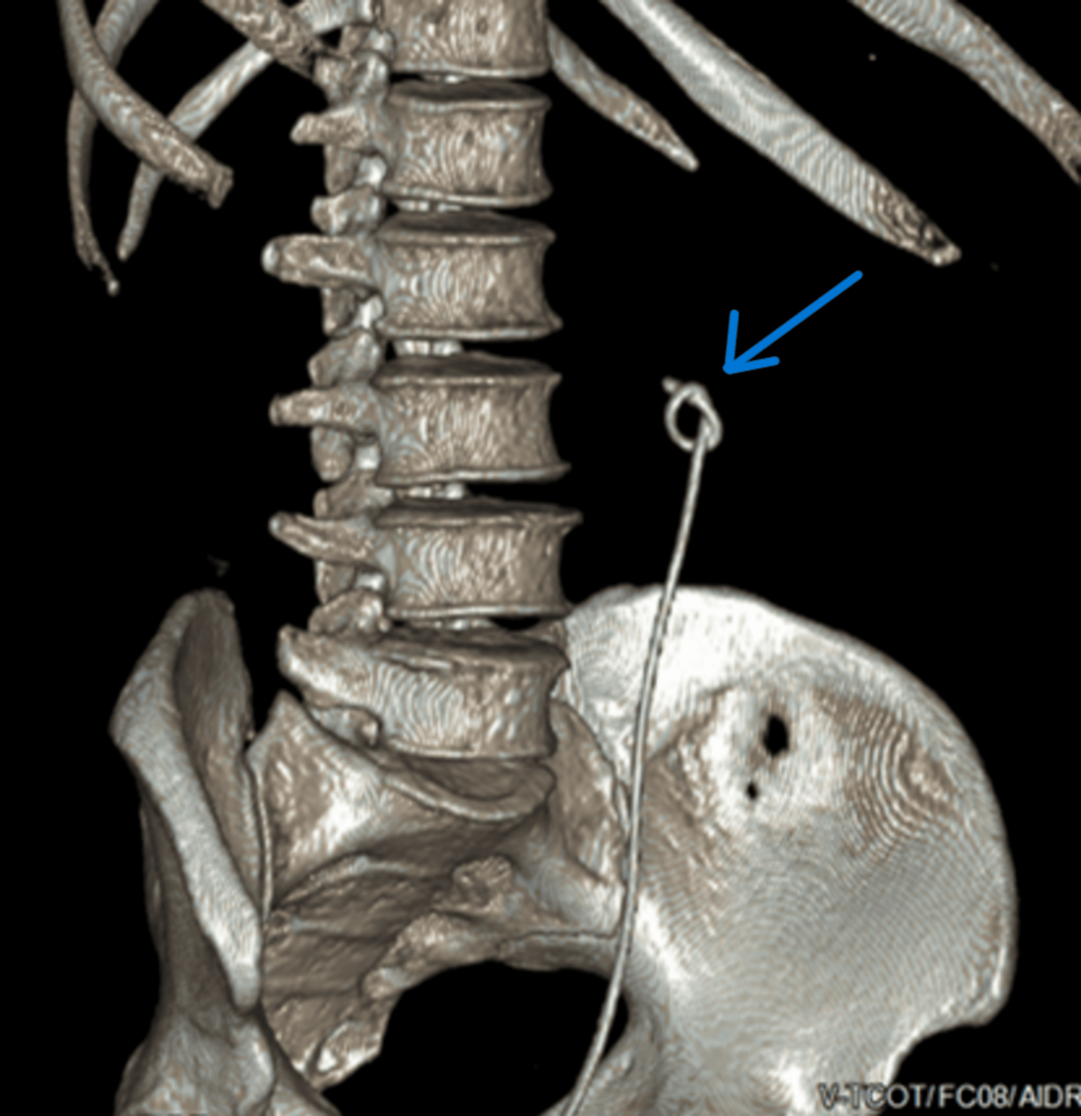
The upper part of the stent can be seen at the proximal ureter outside the renal pelvis and is knotted.

The patient underwent a rigid cystoscopy, and a left double J (DJ) ureteric stent of size 4.8 and a length of 26 cm was inserted under vision and X-ray guidance without difficulties. The patient was then scheduled for a semirigid ureteroscopy after three weeks, but due to the COVID-19 pandemic and the shutting down of elective surgical procedures in the hospital, the patient lost his scheduled booking and presented to the urology clinic after around seven months of stent insertion. CT KUB without contrast was repeated and showed that the lower part of the left DJ ureteric stent had migrated to the proximal part of the anterior urethra with encrustation around the lower part. The upper part of the stent was seen at the proximal ureter outside the renal pelvis and was knotted. The left vesicoureteric stone was not visualized as the patient gave a history that he passed the stone. Left-sided hydronephrosis was also noted (Figures 2, 3).

**Figure 4:**
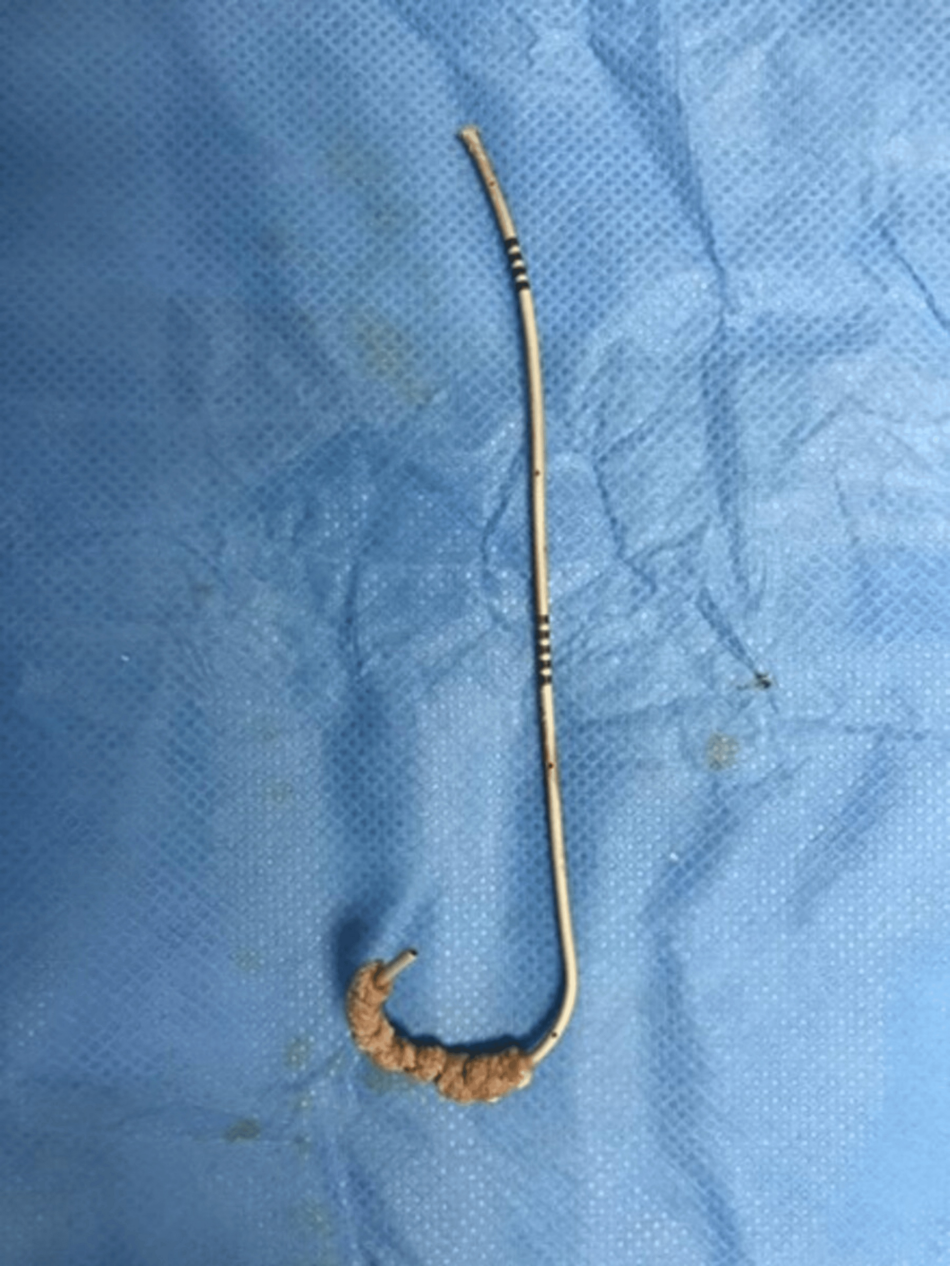
Transection of the stent in the bladder was done using a laser and the distal part of the stent was easily removed with a grasper.

A few days later, the patient was electively admitted and a rigid cystoscopy was performed. The stent was visualized in the posterior urethra with encrustation around its distal part. A trial of stent withdrawal using a grasper was attempted, but resistance was noted, so force was not applied to avoid ureteral injury. Subsequently, a semirigid ureteroscope was inserted in the bladder, and the transection of the stent in the bladder was done using a laser. The distal part of the stent was removed with the grasper easily, and then another left DJ ureteric stent was inserted beside the remaining part of the previous one (Figure 4).

**Figure 5:**
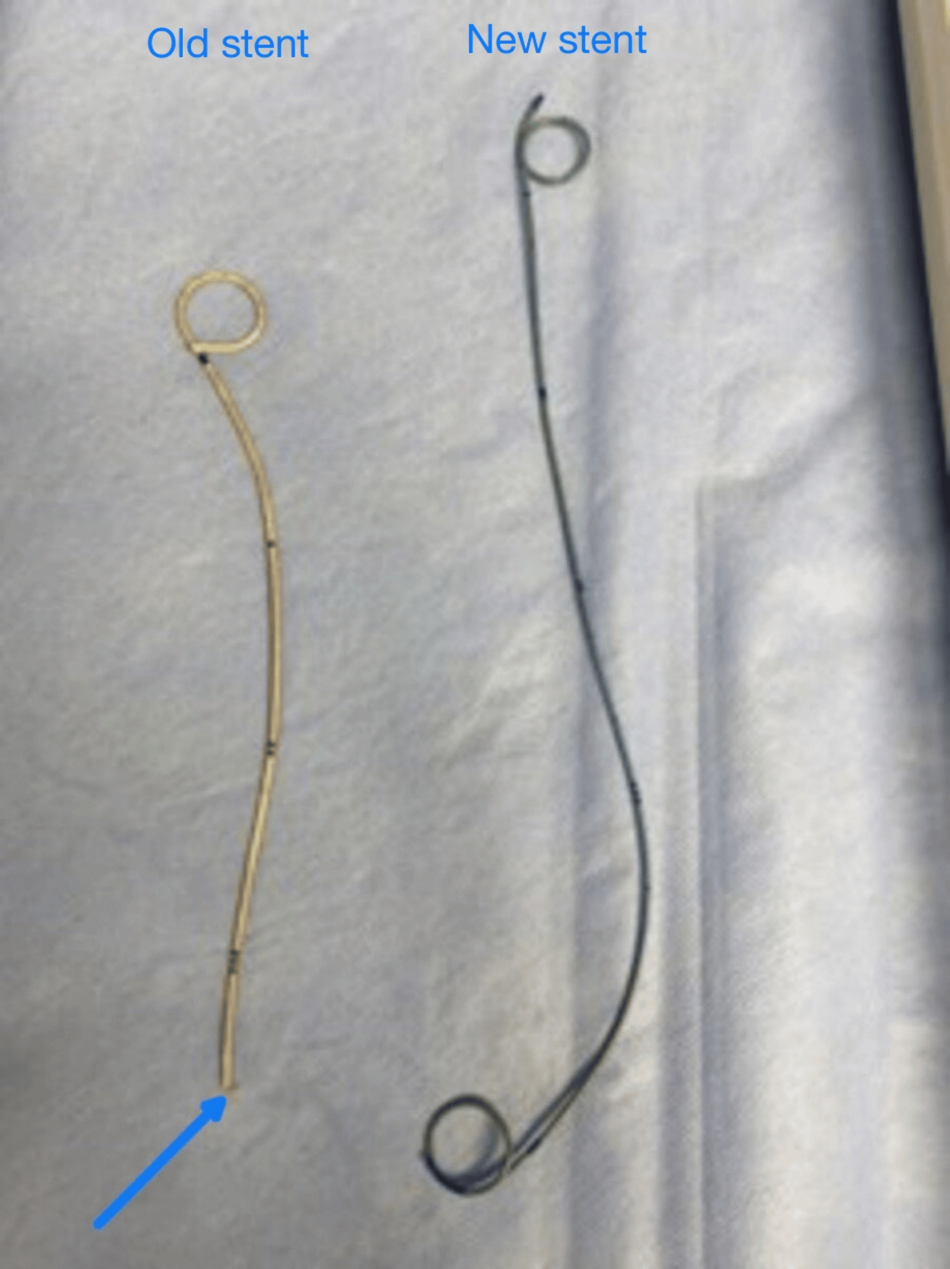
The remaining part of the old stent and the new stent after their removal.

A week after the procedure, the patient underwent one session of ESWL (power: 4-5, frequency: 100 Hz/minute, shock: 2,500 shocks), targeting the knotted part of the DJ ureteric stent. A repeat CT KUB showed the old stent’s lower end had migrated to the distal ureter, the upper end of the stent in the renal pelvis was unknotted, and the new DJ ureteric stent was in a normal position. The patient was re-admitted after one week for removal of both ureteral DJ stents. Removal of the newly inserted stent by rigid cystoscope and grasper was smooth without resistance under vision and X-ray guidance. Afterward, a semirigid ureteroscope was inserted in the left ureter. The remaining part of the old DJ stent was smoothly withdrawn by grasper under vision and X-ray guidance without any resistance (Figure 5).

## Discussion

A tied ureteral stent was initially reported in 1989 [2]. The propensity of the stent to knot inside the ureter is a therapeutic challenge that carries the danger of ureteral avulsion. A knotted ureteral stent can occur at any age. Corbett et al. [8] and Flam et al. [9] documented two cases of individuals, one of whom was four years old and another who was 86 years old. According to the published literature, the gender ratio between men and women is 3:2. Among the various possibilities offered are single-J, DJ, and multiple-length ureteral stents. Unusual consequences of an implanted stent in a ureter include tying, ureteric eroding, fistulae, breakdown, movement, encrustation, and infection [10].

It is unclear exactly what causes the knotting of stents. Since stent sizes ranging from 4.7 to 7 Fr have been linked to knotting. Stents with smaller diameters and flexibility in terms of maneuverability do not appear to increase the risk [11]. Stent encrustation but not knotting may be more likely with a prolonged stent placement period.

The most typical presentation involves issues withdrawing the stent. A standard radiograph might identify tying. Numerous methods for managing this difficult illness have been documented. In around 50% of the documented cases, simple traction proved effective in retrieving stents. Even though traction is an easy procedure, there is a good probability that the knot will become significantly tighter. Following extraction, renal colic and transient hydroureteronephrosis are not unusual [11].

It is important to consider the possibility of ureteric or renal unit damage [12]. If resistance arises upon pulling, another kind of action needs to be looked for. The recommended endourological procedure is typically ureteroscopy. It has been reported that tied stents can be removed effectively via a grasper and a holmium:YAG laser [12]. Retro entry to the proximal knots from the ureter that has a stent in place might not always be smooth. Any moment during the procedure, open ureterotomy, antegrade stents removal, or percutaneous nephrostomy interventions should be planned and ready to go.

In our case, we used an unusual way of removing the knotted ureteral stent. We initially started with simple traction but failed. Subsequently, using a cystoscope, the distal part of the knotted stent was transected, and another ureteral stent was inserted beside the remaining knotted transected stent. After that, a session of ESWL was performed targeting the knot in the proximal part. We believed that applying shock waves may cause the stent’s knotted portion to open, and it succeeded. Several management options have been reported in the literature, including flexible ureterorenoscopy and stent removal and ESWL and percutaneous access to the kidney and stent removal [7,5,11,12]. However, we believe that ESWL is the least invasive and worth trying. If ESWL did not resolve the issue, we had planned to opt for flexible ureterorenoscopy and stent removal. Plain CT was repeated which showed the knotted transected stent distal end migrated to the distal ureter, the proximal end of the stent in the renal pelvis was unknotted, and the new DJ ureteric stent was noted in normal position. The patient was then re-again after one week for the removal of both ureteral DJ stents. The removal of the newly inserted stent by rigid cystoscope and grasper was smooth without resistance under vision and X-ray guidance. The semirigid ureteroscope was inserted in the left ureter, and the remaining part of the unknotted transected stent was smoothly withdrawn by a grasper under vision and X-ray guidance without any resistance.

## Conclusions

This case exemplifies the importance of adaptability in clinical practice, particularly when confronting unexpected complications. The successful outcome underlines the necessity for urologists to be familiar with various management techniques for stent-related complications, including the use of advanced technologies such as ESWL as an adjunctive treatment strategy. Ongoing training in endourological techniques and an understanding of potential complications can significantly improve patient outcomes and reduce the risks associated with ureteric stent placement.
